# Effect of Size and Temperature on Water Dynamics inside Carbon Nano-Tubes Studied by Molecular Dynamics Simulation

**DOI:** 10.3390/molecules26206175

**Published:** 2021-10-13

**Authors:** Amit Srivastava, Jamal Hassan, Dirar Homouz

**Affiliations:** 1 Department of Physics, Khalifa University of Science and Technology, Abu Dhabi 127788, United Arab Emirates; amit.srivastava@ku.ac.ae (A.S.); jamal.hassan@ku.ac.ae (J.H.); 2 Department of Physics, University of Houston, Houston, TX 77030-5005, USA; 3 Center for Theoretical Biological Physics, Rice University, Houston, TX 77030-1402, USA

**Keywords:** carbon nano-tube, water dynamics, molecular dynamics simulation

## Abstract

Water transport inside carbon nano-tubes (CNTs) has attracted considerable attention due to its nano-fluidic properties, its importance in nonporous systems, and the wide range of applications in membrane desalination and biological medicine. Recent studies show an enhancement of water diffusion inside nano-channels depending on the size of the nano-confinement. However, the underlying mechanism of this enhancement is not well understood yet. In this study, we performed Molecular Dynamics (MD) simulations to study water flow inside CNT systems. The length of CNTs considered in this study is 20 nm, but their diameters vary from 1 to 10 nm. The simulations are conducted at temperatures ranging from 260 K to 320 K. We observe that water molecules are arranged into coaxial water tubular sheets. The number of these tubular sheets depends on the CNT size. Further analysis reveals that the diffusion of water molecules along the CNT axis deviates from the Arrhenius temperature dependence. The non-Arrhenius relationship results from a fragile liquid-like water component persisting at low temperatures with fragility higher than that of the bulk water.

## 1. Introduction

Water transport through nano-channels is a fundamental process in many emerging applications, such as drug delivery [1,2], intracellular solute transport [3], cancer therapy [4], and water treatment technologies [5]. Water exhibits different physical properties when confined in nano-scale geometries compared to that of bulk [6,7,8,9,10]. Recent advances in both theoretical and experimental techniques have resulted in noticeable progress in investigating nano-confined water systems. Many of the recent studies have focused on the structure and dynamics of water molecules inside CNTs and their temperature and size dependence. However, understanding the microscopic mechanisms of water dynamics remains elusive [8,11]. Experimental progress has been achieved using different methods, such as infrared spectroscopy [12], Raman spectroscopy [13], neutron scattering [14,15], X-ray Compton scattering [16], and Nuclear Magnetic Resonance [17], to study the structure and dynamics of confined water inside CNTs. However, until now, there has been a limited number of experimental studies at the molecular level exploring how water organizes and diffuses inside the CNTs [18]. Recently, Bernardina et al. [19] used infrared spectroscopy to study water dynamics in single-wall carbon nano-tube (SWCNT) with different diameters, and they observed that water molecules confined in CNT remain loosely bound, even for filled tubes.

Theoretically, most studies have utilized computer simulations to address the pertinent questions of confinement-related effects on the structure and dynamics of water. Hummer et al. [20] have shown that water molecules inside CNT are arranged as a one-dimensional ordered chain that diffuses cooperatively and moves in a pulse-like manner through the nano-tube. Molecular dynamics (MD) simulations of water molecules confined in CNTs of different diameters performed by Pascal et al. [21] revealed that the water inside the CNTs is more stable than in bulk. Moreover, they showed that water dynamics depend on the CNT diameter. This is due to the increased translational and rotational entropy inside sub-nanometer CNTs and increased transnational entropy in larger CNTs. Striolo [22] has conducted several MD simulations for microcanonical ensemble of water molecules confined in CNTs of diameter 1.08 nm using axial periodic boundary conditions. These simulations show that water molecules inside the nano-tube initially undergo fast ballistic diffusion (MSD ∼$t^{2}$) which crosses over to Fickian type diffusion at long times (MSD ∼*t*). Mukherjee et al. [23] confirm that the dynamics of water inside 0.8 nm-CNTs is Fickian in nature, not subdiffusive, despite the one-dimensional chain arrangement of water molecules. Their analysis suggests that such dynamics arise because of the cooperative motion of the confined water molecules due to the strong positional correlations. However, the results from MD simulation studies by Ye et al. [24] suggest that water confined in (6, 6) CNTs exhibits a single-file-type diffusive mechanism that shifts to the Fickian type inside larger CNTs. They further reveal that the diffusion coefficient of the confined water is non-monotonically dependent on the CNTs diameter. MD simulation studies by Farimani and Aluru [25] on CNTs of finite length with bulk regions on both open ends of the CNTs also suggest that the diffusive mechanism of water inside sub-nanometer CNTs is a single file form. They also find that water molecules’ collective diffusion coefficients were enhanced upon increasing the size of CNTs, except for small CNTs between 0.95 nm to 1.20 nm.

Recently [26], we addressed these issues using two-dimensional NMR diffusion- relaxation $D-T_{2eff}$ spectroscopy and we found that the water molecules inside the nano-tube resolve in two or more tubular components acquiring different-self diffusion constants (*D*) for a specific CNT diameter. Moreover, for the first time [27], we reported experimental results on the stratified water diffusion in CNTs and showed that water dynamics at the center of CNT exhibiting non-Arrhenius behavior, characterized by ultrafast diffusion and extraordinary fragility.

Many MD simulation studies were devoted to investigate diffusion of confined water molecules inside CNTs of different sizes. Most of these studies have been limited to room temperature. Therefore, the effect of temperature on water diffusion inside CNTs has not been extensively explored yet. In this study, we performed MD simulations to study water flow inside several CNT systems. The CNTs considered have the same length of 20 nm, with diameters ranging 1.0 nm to 10 nm. The simulations were conducted at different temperatures, ranging from 260 K to 320 K. The primary goal of this study is to determine the nature of the diffusion inside CNTs and how the temperature affects the diffusion and ordering of water molecules.

## 2. Materials and Methods

MD simulations have been performed to investigate the diffusion of water molecules inside SWCNT of different sizes. Different systems were simulated for CNTs with different diameters. We have investigated the armchair carbon nano-tube, namely (n, n), where n = 8, 15, 18, 22, 26, 37, and 73, of diameters 1 nm, 1.4 nm, 2.0 nm, 3.0 nm, 3.5 nm, 5.0 nm, and 10.0 nm, correspondingly.

### Simulation Setup

**Figure 1:**
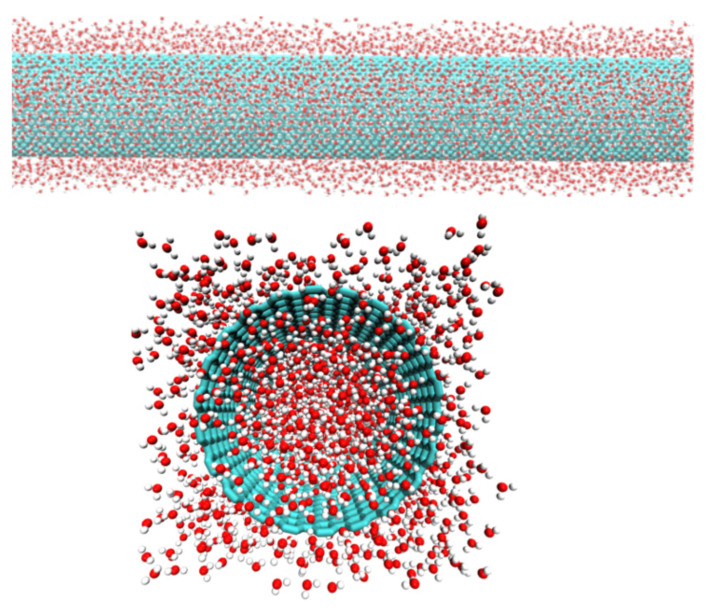
Schematic diagram of a water-CNT system used in simulations: (**upper**) Side view and (**lower**) perspective top view.

All the MD simulations were performed using NAMD [28]. A schematic diagram of water inside SWCNT is shown in Figure 1. Water molecules in the simulations were represented using the Simple Point Charge/Extended (SPC/E) model [29], which is known to accurately predict many of the bulk properties of water. The non-bonded interactions between the carbon atoms were modeled using Lennard–Jones Potential. We choose the parameters ϵ = −0.069 kcal/mol and $r_{min}$ = 3.805 Å, given by Werder et al. [30]. The CNTs were kept rigid by fixing the positions of the carbon atoms throughout the simulations. The water-CNT systems were investigated in the temperature range from 260 K to 320 K. The temperature was set to the target value using the Langevin thermostat. The pressure was maintained at 1.0 atm using Nose-Hoover Langevin piston with a period of 100 fs and a damping time scale of 50 fs. Unlike most previous studies that assumed axial boundary conditions along the CNT axis, we allowed water molecules to flow in and out of CNTs with finite length. Long-range electrostatic interactions were computed by particle mesh Ewald summation method (PME). The simulation integration time step was 2 fs. Bonded interactions were calculated every time step, while non-bonded interaction was calculated every two steps, with a cutoff of 12 Å and switching function of 10 Å. All simulated systems were minimized for 10,000 steps, then gradually heated to the target temperature, and then equilibrated at this temperature for 50,000 steps (100 ps) before the production runs. The production simulations were run for a total time of 50 ns. The system configuration was saved every 500 steps (1 ps) for analysis.

Firstly, we calculated the radial density [31] profile for water inside each simulated CNT system in order to elucidate the ordering of water molecule. The radial density is calculated by segmenting the volume inside the CNT into concentric cylindrical shells and averaging the number of oxygen atoms in each shell during the simulation. The radial density is defined as:

(1) $$\rho_{radial}=\frac{M}{\pi l{\left(d_{eff}/2\right)}^{2}},$$

where *M* is total water mass confined inside the CNT, and *l* is the CNT length. $d_{eff}$ is the effective diameter of CNT defined as: $d_{eff}$ = $d-r_{min}$, where *d* is the diameter of CNT, and $r_{min}$ is the Lennard–Jones (LJ) parameter for carbon-oxygen interaction.

To characterize the structure of water molecules inside CNT, we also calculated the number of hydrogen bonds (HBs) per water molecule. This number was computed using the following geometrical criteria:

α ≤ 30∘

|**r**_*OO*_| ≤ 3.50 Å,

where α is the OH···O angle, and $|r_{OO}|$ is the distance between two oxygen atoms.

In addition, we calculated oxygen density maps obtained by dividing the correspondent plane in square bins of 0.1 Å length and then counting the number of oxygen atoms in each square. Higher oxygen densities are represented in red, while low oxygen densities are represented in blue color.

Due to the confined geometry, the diffusion of water is minimal along the radial direction, and it is almost zero [32]. Therefore, here, we consider only the axial self-diffusion coefficient (*D*) along the z-direction. The value of *D* was determined using mean squared displacement function (MSD) in the axial direction. The MSD of water molecule inside the nano-tube was calculated using

(2) $$\langle \Delta z^{2}\left(t\right)\rangle =\frac{1}{N}\sum_{i=1}^{N}{\langle {\left[z_{i}\left(t+t^{\prime }\right)-z_{i}\left(t^{\prime }\right)\right]}^{2}\rangle }_{t^{\prime }},$$

where *t* is the time difference, $t^{\prime }$ is a time origin, and *N* is the number of water molecules. MSD was calculated over a time interval of 1.0 ns at a sampling rate of 1.0 ps. MSD was then averaged over 50 such time intervals. The interval length, 1.0 ns, was chosen carefully to give water molecules enough time inside CNTs before exiting. In order to estimate the diffusion constant *D*, we used our recent proposed algorithm [33], which is capable of fitting the MSD function with different regimes corresponding to different time scales. The algorithm fits the MSD to a continuous piece-wise function and finds the breakpoints which separate different modes of motion. The diffusion coefficients were calculated for all of the water inside the CNT and for all the components obtained from the density profile.

## 3. Results and Discussion

**Figure 2:**
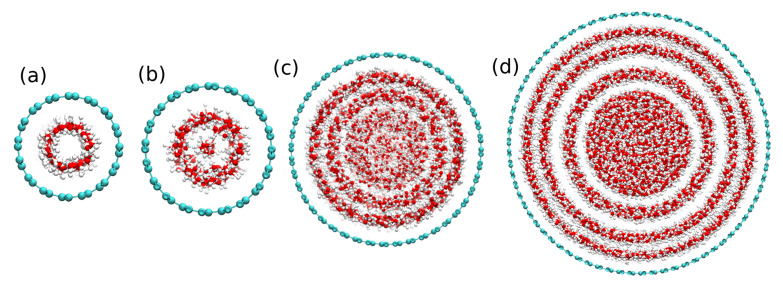
Atomic distribution of water molecules inside CNTs of different sizes: (**a**) 1.0 nm, (**b**) 1.4 nm, (**c**) 3.0 nm, and (**d**) 5.0 nm. The green color corresponds to carbon atoms, red color to oxygen atoms, and the white color to the hydrogen atoms. Circles represent the stratified layers of water inside CNT channels.

The organization of water molecules inside CNTs varies, depending on their confinement sizes. It forms a 1D polymer-like chain of water molecules in ultra-narrow CNT diameter (less than 1.0 nm). However, for CNTs with larger diameters, more water molecules fill the space inside the CNT and organize in the coaxial tubular sheets. Figure 2 shows the snapshots of water ordering for four CNT systems with diameters 1.0, 2.0, 3.0, and 5.0 nm in simulations performed at room temperature (300 K).

In all cases, water molecules inside CNT channels are shown to arrange in concentric water tubular (CWT) sheets (circles in the snapshot), in agreement with the previous work (see, for example, Reference [8] and references therein). The number of CWT sheets inside the CNT channels depends on the size of the CNTs, oxygen–oxygen, and oxygen-carbon interactions. It is also observed that the layered water arrangement into CWT sheets becomes denser upon increasing the CNT size. By continuing to increase the CNT size, the dynamics of water molecules will gradually approach the bulk limit. This layered structure of water molecules in the simulated systems does not depend on the temperature.

**Figure 3:**
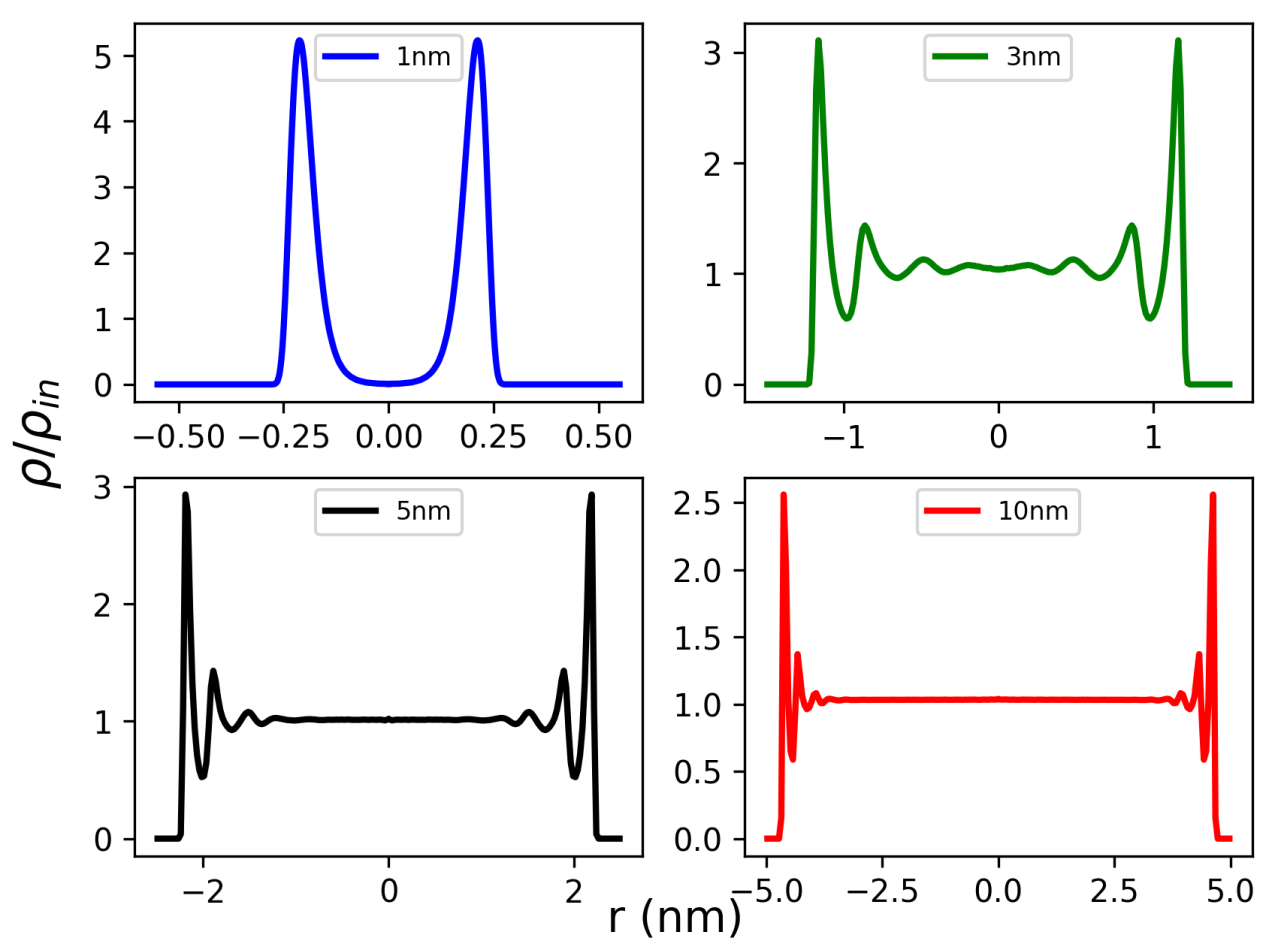
Radial water local density inside different CNT sizes at room temperature: (**a**) 1.0 nm, (**b**) 3.0 nm, (**c**) 5.0 nm, and (**d**) 10 nm. The *x* axis shows the inner diameter of CNT, whereas zero represents the center of the nano-tube.

The change in the water density profile is the most evident effect of confinement inside CNTs. The radial density is strongly affected by the stratified structure of the water molecules as described above. Figure 3a–d show the radial density profiles inside the CNTs of different diameters at room temperature. These figures show that water is well structured for all nanopore radii. Figure 3a shows the density profile of water of CNT size of 1.0 nm. The single peak in the density profile indicates that water forms a single tubular layer, in agreement with the previous work [34]. Due to the hydrophobic interactions, water molecules are repelled away from the CNT carbon atoms by a distance of about 0.3 nm.

As the diameter of CNT increases, additional water layers are formed corresponding to the additional density peaks (see Figure 3b–d). For large CNTs, a bulk-like density profile is observed near the nano-tube center in agreement with previous studies [8,34]. For the 3.0 nm-CNT, we observed two additional water layers consistent with the NMR experiments [26]. Density maps corresponding to the oxygen occurrence during MD simulation in xy direction are shown in Figure S1 (Supporting Information (SI)). The density colormaps confirm the layered tubular sheet structure of water inside CNTs.

**Figure 4:**
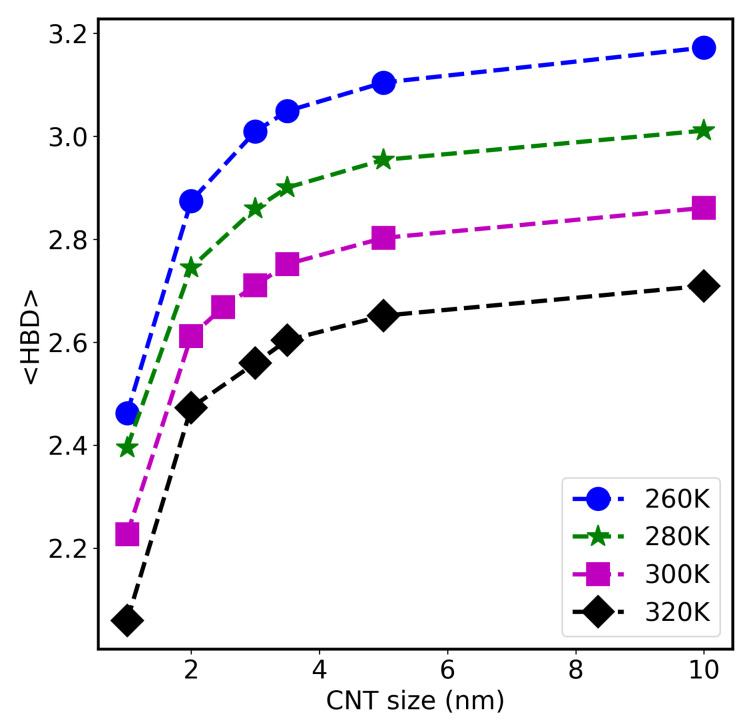
Average number of hydrogen bonds per water molecule versus CNT sizes at different temperatures (260 K, 280 K, 300 K, and 320 K).

In order to examine the structure of the confined water, the average number of hydrogen bonds (HB) per water molecule is calculated. These numbers were obtained for water-CNT systems of sizes ranging from 1.0 nm to 10 nm at different temperatures (from 260 K to 320 K), and results are shown in Figure 4. As seen, the number of HB is highly related to the size of the systems and their temperature. Upon increasing temperature, the number of HB decreases, and water molecules get less structured. Due to the geometrical restrictions (and also rigidity that will be discussed later), the allowed numbers of HB is smaller in small CNT sizes than those at larger CNT systems. For example, at 300 K, the number of HB per water molecule in 1.0 nm is 2.2, while it increases to 2.8 in the 10-nm CNT system. Similar results at room temperature were reported previously [8,25]. Another observation is that the number of HBs in the systems discussed here is lower than the bulk phase (3.7 at room temperature) [25,35]. These differences are more noticeable between small CNT systems and bulk water (2.2 in 1.0 nm CNT system compared to 3.7 in bulk water). Upon increasing temperature, the hydrogen bonds among water molecules break, and the radial density peaks near the CNT walls also deceases (as shown in Figure S2 for 1.0 nm CNT size). On the other hand, the axial density distributions of water along the *z*-direction in the CNT systems studied here were observed to remain unchanged with sizes and temperatures (see Figure S3 in SI). This shows that water molecules are uniformly distributed along the *z*-axis.

To get more insight into the effect of HBs and molecules’ structure on their mobilities, we have studied diffusion modes of water inside different CNT systems as a function of temperature. As we will see soon, there is a direct connection between water structure and its diffusion. In addition, different confinement sizes leads to multiple diffusion modes.

**Figure 5:**
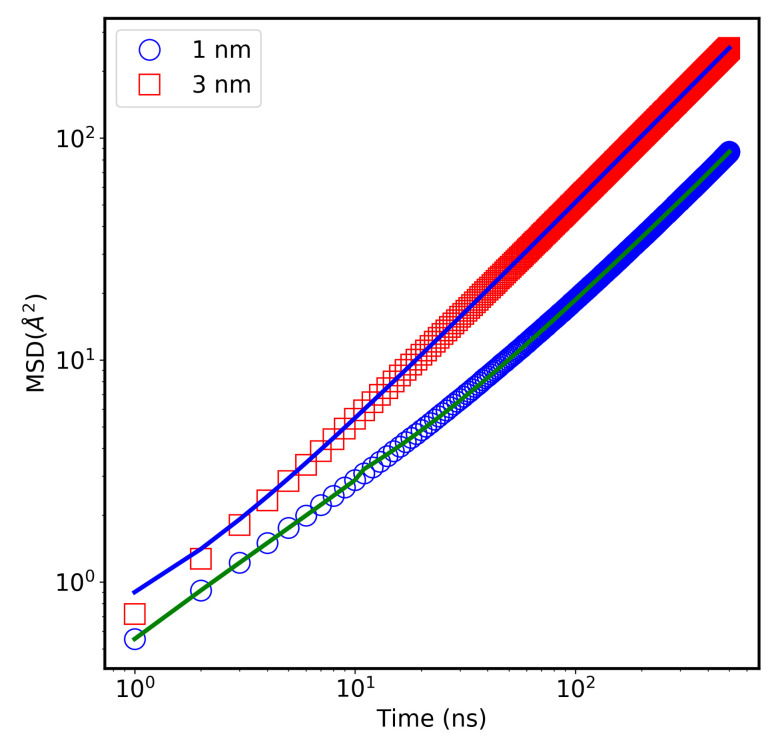
Mean square displacement (MSD) as a function of time of water inside CNT at T = 300 K calculated using MD simulation. The solid line is fitted data using Equation (7) in Reference [33].

Based on our fitting algorithm [33] of the MSD function, we can identify different diffusion regimes in terms of a scaling factor (power) (α). The value of this power, α, is one for Fickian processes and larger (or smaller) than one for superdiffusive (or subdiffusive) systems. Figure 5 shows MSD curves as a function of time for CNTs with diameters of 1.0 nm and 3.0 nm. The data and the fitted curves show α = 1, indicating a Fickian diffusion behavior for both sizes. Further information on the temperature dependence of MSD for water molecules in 3.0 nm CNTs, at different temperatures, is shown in SI (Figure S4). The data were successfully fitted with the model obtaining α = 1, confirming the Fickian nature for the diffusion at all the temperatures.

**Figure 6:**
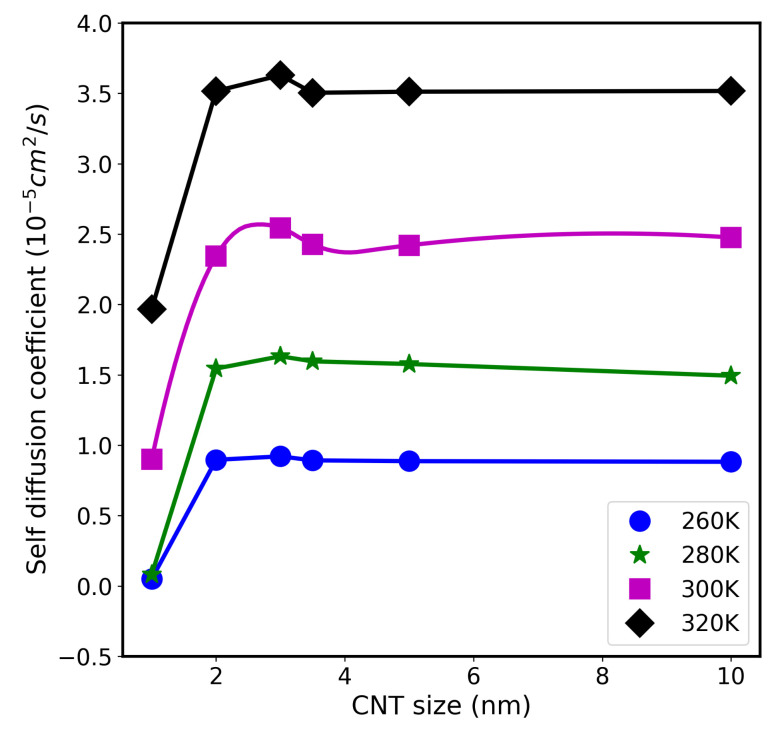
Self diffusion coefficient versus CNT sizes, at different temperatures (260 K, 280 K, 300 K, and 320 K).

The axial diffusion coefficients of water molecules for all CNT system were calculated using our fitting algorithm [33]. Figure 6 shows these results at different temperatures (260 K, 280 K, 300 K, and 320 K). As expected, the values of *D* increase with temperature. For example, the value of *D* for 3.0-nm system increases from ∼0.921 ×$10^{-5}$${\mathrm{cm}}^{2}/s$ at 260 K, to 1.632 ×$10^{-5}$${\mathrm{cm}}^{2}/s$ at 280 K, to 2.744 ×$10^{-5}$${\mathrm{cm}}^{2}/s$ at 300 K, and to 3.629 ×$10^{-5}$${\mathrm{cm}}^{2}/s$ at 320 K. This is due to the change of water structure (reduction of the number of HB per water molecules), as discussed earlier within the text related to the result presented previously in Figure 4.

Furthermore, the values of *D* inside the small CNT (diameter of 1.0 nm) is 0.9 ×$10^{-5}$${\mathrm{cm}}^{2}/s$ at 300 K, whereas, at 260 K and 280 K, its value is quite small (∼0.05 ×$10^{-5}$) ${\mathrm{cm}}^{2}/s$. In this case, water molecules are frozen inside the CNT channels in the tubular-shape shown as a ring in Figure S1a. In addition, each water molecule is attached, on average, to 2.45 other water molecules through hydrogen bonds, as shown in Figure 4. The slow dynamics of water in this small confinement is due to the rigidity of water molecules through the formation of an organized network depicted as a tubular shape shown in Figure S1, considering that the cutoff length of an hydrogen bond is about 0.35 nm [32].

For CNT diameters above 2.5 nm, the axial diffusion coefficient approaches the bulk value (∼2.5 ×$10^{-5}$${\mathrm{cm}}^{2}/s$) at room temperature. These results agree with previous study which showed that water with more than two layers loses memory of the CNT wall and tends to acquire the bulk water structure [8]. Therefore, in large CNT nanopores, the structure of the water (near the center) and its hydrogen bond network resembles bulk water. For intermediate CNT sizes, diameter (∼3 nm), we obtained the highest *D* value of 2.74 ×$10^{-5}$${\mathrm{cm}}^{2}/s$ at room temperature. A careful inspection of the data presented in Figure 4 shows no dramatic reduction in the number of HBs of water inside 3.0 nm CNT, compared to other systems. Therefore, the enhancement of water diffusion in this particular confinement size cannot be totally attributed to change in the water orientation or structure. Recently, Gkoura et al. [26] also observed the enhancement in water diffusion for CNT with length 3.5 nm using the two-dimensional nuclear magnetic resonance diffusion-relaxation (*D*-$T_{2eff}$) spectroscopy method. The observed diffusion coefficient measurements agree qualitatively with our MD simulation results. Similar results were obtained by MD simulations [25,34,36] and experimental groups [37,38]. However, the underlying diffusion enhancement mechanism is still not very clear. One possible explanation for maximum and minimum diffusion rates observed in CNTs of different diameters is related to this competition between the water-wall contact area and the volume occupied by the water.

**Figure 7:**
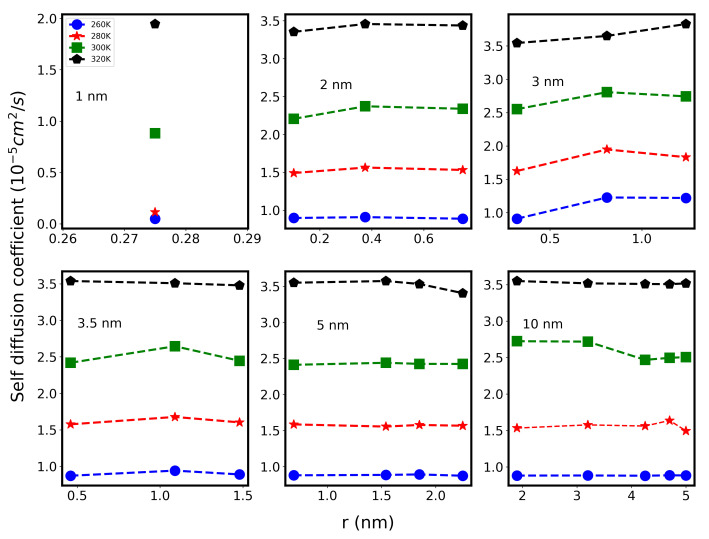
Self diffusion coefficient of different water shells (shown in Figure 1) inside CNT with different diameters. The *x* axis shows the distance of the water shell from the center of CNTs. Blue, red, green, and black color data points correspond to temperature 260 K, 280 K, 300 K, and 320 K.

As we have already shown that water molecules inside the CNT channel of different diameters are arranged in concentric water tubular CWT sheets (Figure 1), it would be important to examine the dynamic of water in each of these CWT sheets. Figure 7 shows the results of Ds of different water sheets inside various CNT systems at temperatures of 260 K, 280 K, 300 K, and 320 K. We observe striking differences in water dynamics between the CWT sheets, in some CNT sizes. At room temperature, water *D* values inside 2.0 nm and 5.0 nm CNT channels remain almost the same for all the sheets. On the other hand, inside 3.0 nm, 3.5 nm, and 10 nm, it was observed that water diffuses faster in the central sheets, compared to others. These results were experimentally verified by our group using NMR spectroscopy [26]. Structural and vibrational studies [39,40] have also shown that water confined inside a CNT of size 3.0 nm acquires ice-like clusters, showing cooperative motion with high diffusion. In this work, water clusters can diffuse smoothly and fast into these nanopores. The physical reason for this fast diffusion was attributed to the cooperative movement of water molecules as long as they are far from the hydrophobic effects of the CNT walls and radial interactions with other molecules in the other sheets. Moreover, further analysis of CTW sheets show that water has its smallest average number of HBs in the inner shells for the nano-tube with size 3.0 nm (Figure S5). This observation is consistent with the increased mobility of water near the axis of the CNT for this size.

**Figure 8:**
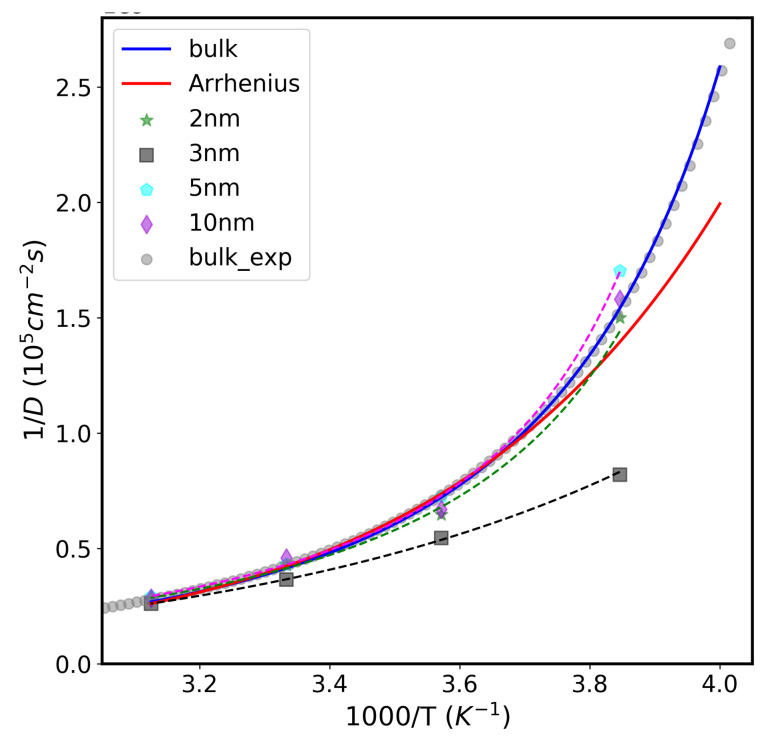
Self diffusion coefficient reciprocal (1/*D*) versus 1000/T of the water confined inside nano-tube of different sizes. The blue line is the theoretical 1/*D* versus 1000/T curve for bulk water plotted using Speedy–Angell power law function. The red line is the theoretical 1/*D* versus 1000/T curve of an ideal liquid obeying Arrhenius law. The dashed lines are power law fits of the self diffusion coefficient data of different CNT sizes.

Finally, we are going to address the puzzling transition that water undergoes from fragile to strong liquid. In certain liquids, denoted as “strong-liquids”, temperature dependence of some thermodynamic properties, such as self-diffusion coefficients, exhibit an Arrhenius behavior, whereas, in other liquids, categorized as “fragile-liquids”, these properties deviate from that law. Water acts as a fragile liquid at ambient temperature, while appearing as a strong-liquid upon super cooling [41]. We have examined the fragility of water molecules inside different CNT sizes and various temperatures. Figure 8 shows the temperature dependence of the inverse of the self diffusion coefficient 1/*D* versus 1000/T of the water confined in different CNT sizes (from 2.0 nm to 10 nm) in the temperature range between 260 K to 320 K. Experimental data for bulk water and their fitted curve was taken from our previous paper [26]. The bulk data was fitted to the Speedy–Angell power law (shown in blue color), i.e., *D* =$D_{0}$${\left(\left(T/T_{S}\right)-1\right)}^{\gamma }$, where $D_{0}$= 1.635 ×$10^{-8}$, $T_{S}$ = 215 K, and γ= −2.063. The red line shows the Arrhenius curve for ideal liquid obeying the Arrhenius law: *D* = $D_{0}$$exp(-U/k_{B}T)$, inserted inside the figure for comparison. As seen, at high temperatures, bulk water follows the Arrhenius law. However, at low temperature ∼271 K, the data strongly deviate and adopt a non-Arrhenius behavior. This crossover behavior has been observed in many glass-forming systems [42].

The diffusion data for confined water in the CNT systems were successfully fitted to the power law, indicating their non-Arrhenius behavior similar to that of bulk water. The parameter γ in Speedy–Angell power law is associated with the fragility and the HB structure which, in turn, is related to the dynamical behavior of water. In small and large CNT sizes (2.0 nm, 5.0 nm, and 10 nm), the nano-tube water dynamics are similar to the bulk water, and the γ value is in the range of −1.9 to −2.1. In 3.0 nm CNT size, water molecules’ D values deviate from the bulk water where the γ value is −3.1. This indicates the high fragility of water molecules in this system. The observed phenomena are consistent with the previous studies [26,27,41].

## 4. Conclusions

This study presents the Molecular dynamics simulation results of water confined inside CNTs of different sizes and at different temperatures. We found that the water is arranged in a 1D chain-like manner in ultra-narrow CNTs. For larger CNT sizes, the water molecules adopt a shell structure and organize in a coaxial tubular sheets. Furthermore, the number of tubular sheets increases with the CNT diameter. CNTs are hydrophobic in nature, and water molecules tend to avoid the contact with the CNT surface. This fact plays a major role in water diffusion in the narrow nano-tubes. Our simulations show a clear enhancement in water diffusion for a favorable CNT size range (3.0–4.0 nm). Moreover, the temperature effects on water dynamics add a new perspective that could explain this enhancement. The results of the simulations show that increasing the temperature will weaken the HB network of confined water and increase the diffusion enhancement. Furthermore, the temperature dependence of the axial diffusion constant of water points to a structure at its most fragile state in the CNT size range (3.0–4.0 nm) These results point to a plausible cause for diffusion enhancement in this size range. The liquid fragility is probably associated with hydrogen bond lifetimes within the carbon nano-tubes. We also observe that, in this size range, the water molecules diffuse faster in center compare to the outer shell. This points to more fragility near center compared to the outer shell. Our simulation results are qualitatively consistent with the experimental NMR results [18], although the time scale in molecular dynamics simulations is much shorter compared to that in NMR experiments.

## Data Availability

The data of this study are available upon request from the authors.

## Supplementary Materials

The following are available online. Figure S1: Density map of water inside nanotube, Figure S2: Temperature dependence of radial water density, Figure S3: Axial water density inside different CNT sizes, Figure S4: MSD at different temperature and Figure S5: Average number of hydrogen bonds per water molecule in the inner shell.
