# Supplementary material for: Effect of Size and Temperature on Water Dynamics inside Carbon Nano-Tubes Studied by Molecular Dynamics Simulation
Source: Molecules. 2021 Oct 13;26(20):6175. doi: 10.3390/molecules26206175 (PMC8540028; doi:10.3390/molecules26206175)
Supplement: Supplementary file 1 [file molecules-26-06175-s001.zip › molecules-1415935-supplementary.pdf]

Article

Supplementary Information for Effect of size and temperature on water dynamics inside the carbon nanotubes studied by molecular dynamics simulation.

Amit Srivastava [1], Jamal Hassan [1] and Dirar Homouz [1,2,3]*

1 Department of Physics, Khalifa University of Science and Technology, 127788 Abu Dhabi, UAE
2 Department of Physics, University of Houston, Houston, Texas 77030-5005, USA
3 Center for Theoretical Biological Physics, Rice University, Houston, Texas 77030-1402, USA
* Correspondence: dirar.homouz@ku.ac.ae

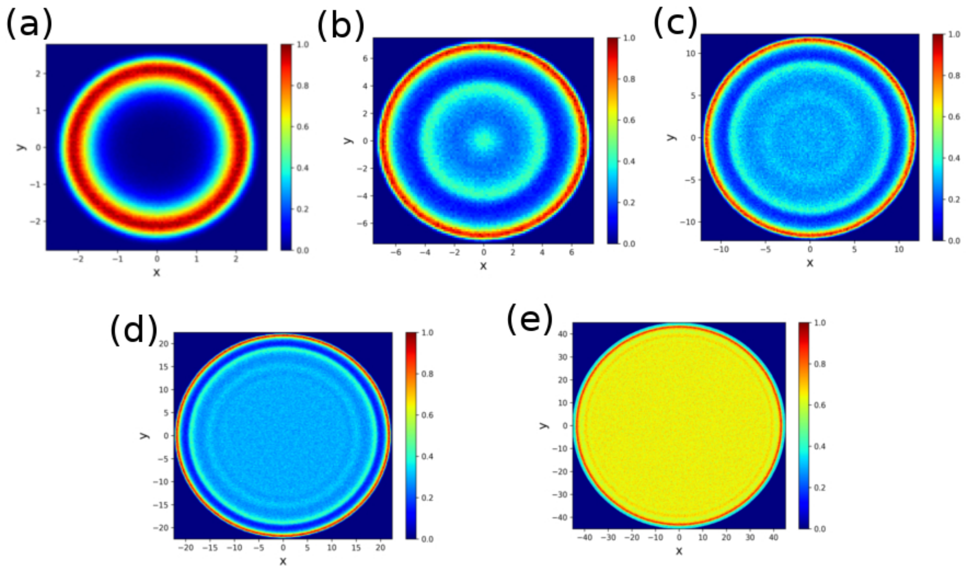

Fig. S1. Density map of water inside various nanotube sizes, shown on the xy plan (a) 1.0 nm, (b) 2.0 nm, (c) 3.0 nm, (d) 5.0 nm, and (e) 10 nm. High water densities are represented in red color, while low water densities tend to have dark blue color.

Citation: . *Preprints* , , .

Received:
Accepted:
Published:

Publisher's Note: MDPI stays neutral with regard to jurisdictional claims in published maps and institutional affiliations.

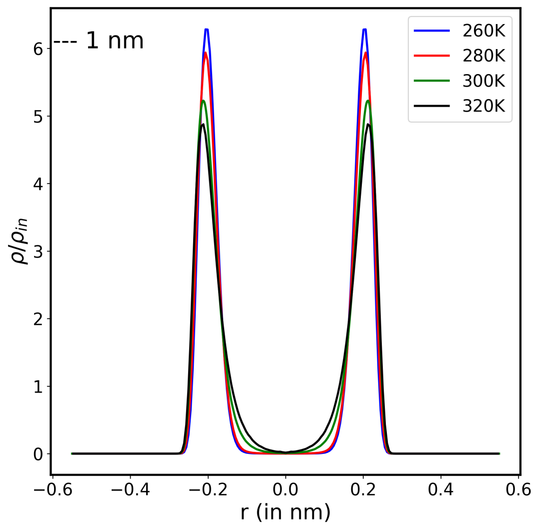

Fig. S2. Radial water local density inside CNT size 1.0 nm at different temperatures. The x axis shows the inner diameter of CNT, whereas zero represents the center of nanotube.

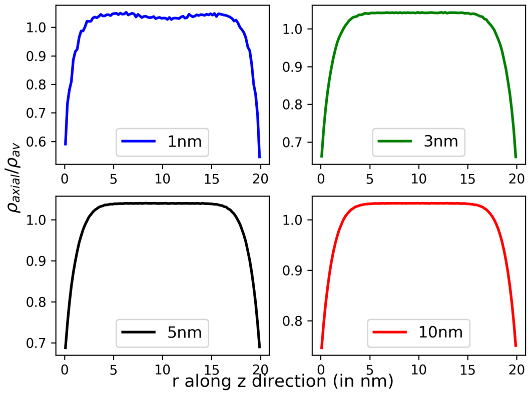

Fig. S3. Axial water density inside different CNT sizes at room temperature. The x axis represents the length of CNT in nm.

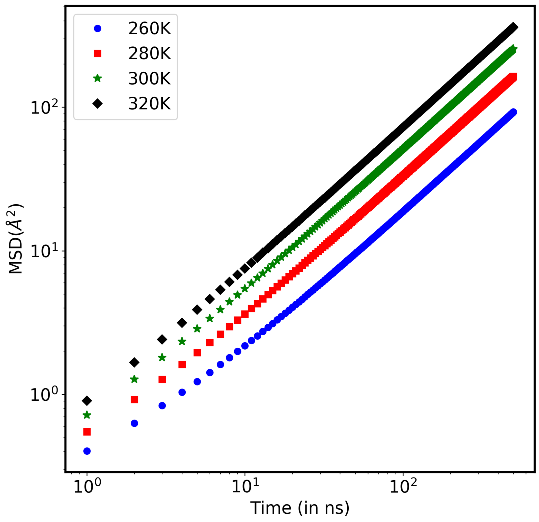

Fig. S4. Mean square displacement (MSD) as a function of time for water inside 3.0 nm CNT, at various temperatures.

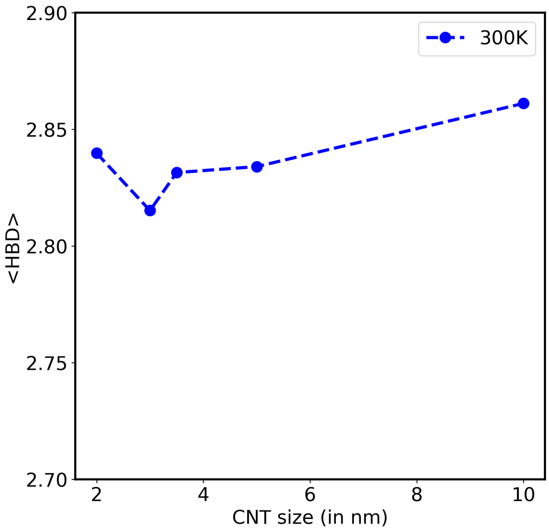

Fig. S5. Average number of hydrogen bonds per water molecule in the inner shell versus different CNT sizes at 300K.
